# The clinical impact of herpesvirus testing on multiplex PCR panels in a pediatric population

**DOI:** 10.1017/ash.2025.10242

**Published:** 2025-11-25

**Authors:** Caitlin Naureckas Li, Cecilia Thompson, Brittany Hunter, Elizabeth Dobler, Natalie Jachym, Emaan Mohsin, Marcelo Malakooti, Lisa Akhtar

**Affiliations:** 1 Division of Pediatric Infectious Diseases, Department of Pediatrics, Ann & Robert H Lurie Children’s Hospital, Chicago, IL, USA; 2 Northwestern University Feinberg School of Medicine, Chicago, IL, USA; 3 Department of Pathology and Laboratory Medicine, Ann & Robert H Lurie Children’s Hospital, Chicago, IL, USA; 4 Division of Hospital-Based Medicine, Department of Pediatrics, Ann & Robert H Lurie Children’s Hospital, Chicago, IL, USA; 5 Division of Critical Care Medicine, Department of Pediatrics, Ann & Robert H Lurie Children’s Hospital, Chicago, IL, USA

## Abstract

The use of multiplex polymerase chain reaction (PCR) panels for diagnosis of clinical syndromes is rapidly growing despite limited data on optimal use cases. We retrospectively reviewed the clinical impact and consequences of the inclusion of herpesvirus targets on the meningitis/encephalitis PCR panel.

## Introduction

A variety of multiplex molecular panels are now commercially available to detect a defined set of organisms associated with a clinical syndrome such as meningitis/encephalitis (ME), gastroenteritis, or pneumonia.^1^ However, these panels often include targets that may not be clinically significant in every patient. Detected organisms may represent normal flora, colonizing organisms, or reactivation of latent viruses in the setting of another underlying illness, particularly in immunocompetent patients.^2^ Positive result interpretation can be challenging, and clinicians may be hesitant to dismiss a positive result.

The multiplex polymerase chain reaction (PCR) ME panel from BioFire (BioMérieux, Salt Lake City, UT) received U.S. Food and Drug Administration clearance in 2015 and contains 14 bacterial, viral, and fungal targets.^3^ Reported positive predictive values (PPVs) vary widely but have been reported to be as low as 37%.^4,5^ Concerns around PPVs are particularly significant in children, where the epidemiology of meningitis differs from adult counterparts; for example, pediatric cryptococcosis is significantly more rare than the same disease in adults.^6,7^ Additionally, the clinical spectrum of meningitis and encephalitis is broad, with limited overlap in presentation between pathogens included on the panel. Given this, we sought to understand the clinical impact and describe potential benefits and harms associated with detection of herpesviruses on the ME panel at our institution.

## Methods

This work took place at our freestanding Midwestern children’s hospital. At our site, an ME panel is typically ordered upfront, without restrictions, any time a sample is collected by lumbar puncture for infectious indications regardless of clinical presentation. Clinicians are encouraged to send a dedicated herpes simplex virus (HSV) PCR as the first line test when this diagnosis is suspected given concerns regarding lower ME panel sensitivity compared to targeted PCR.^8^

We retrospectively reviewed ME panel results from January 2019-January 2025 positive for any virus in the Herpesviridae family (cytomegalovirus [CMV], HSV1, HSV2, human herpesvirus 6 [HHV6], and varicella zoster virus [VZV]). Two infectious diseases attendings (CNL, LA) evaluated clinical significance of results based on documentation by the primary team. We applied descriptive statistics as appropriate. This work was deemed exempt from full review by our local institutional review board.

## Results

1,598 ME panels were performed during the study period from 1,449 individual patients. Thirty-one panels were positive for a total of 32 herpesviruses: 20 HHV6, and 3 each CMV, HSV1, HSV2, and VZV. Five panels were positive for more than one organism: one with two herpesviruses and the remainder with a herpesvirus and a bacterium. Of the 32 positive herpesvirus ME panel results, six (19%) results were determined to have a positive impact on management, five (16%) a negative impact, one (3%) an unclear impact, and 20 (63%) no impact (Table 1). Half of panels with a positive impact on patients (3/6) served as confirmation of central nervous system (CNS) involvement, specifically in patients with known varicella. Two of the remaining three panels with a positive impact were in immunocompromised patients. All positive VZV results resulted in a positive change of management, and all positive CMV results resulted in unnecessary interventions.

**Table 1.** Clinical details of positive tests

Sample number	Positive ME panel herpes virus target	Patient age	Clinical scenario	Known immunocompromising condition?	CSF WBC count (/mm^3^)	Impact of detected herpes virus on other clinical management
1	CMV	2 months	Fever and concern for seizure	No	9	Negative - underwent work-up for congenital CMV, treatment ultimately deemed to not be necessary
2	CMV, *Streptococcus agalactiae*	2 weeks	Fever and positive blood culture	No	6 231	Negative—underwent work-up for congenital CMV with no clear evidence of symptomatic CMV disease Treated with valganciclovir in shared decision making with family. Treated for *Streptococcus agalactiae,* which was also identified on traditional culture
3	CMV, *Escherichia coli*	1 week	Fever and positive blood culture	No	Not performed	Negative - underwent work-up for congenital CMV, treatment ultimately deemed to not be necessary Treatment of CMV ultimately deemed to not be necessary. Treated for *E. coli* meningitis.
4	VZV	7 years	Skin manifestations of VZV and seizures in immunocompromised patient	Yes (autoinflammatory condition)	9	Positive - CNS involvement of VZV confirmed and acyclovir dosing optimized
5	VZV	15 years	Skin manifestations of VZV, clinical concern for meningoencephalitis	No	236	Positive - CNS involvement of VZV confirmed and acyclovir dosing optimized
6	VZV, HHV6	1 week	Skin manifestations of VZV	No	5	Positive (VZV) - CNS involvement of VZV confirmed, acyclovir continued for meningoencephalitis course None (HHV6) - HHV6 not considered in medical decision making
7	HHV6	15 years	Headaches and confusion	No	101	None
8	HHV6	8 years	Fever and seizure	No	3	None
9	HHV6	9 years	Seizure vs rigors	No	<1	None
10	HHV6	7 years	Multiple neurologic changes	Yes (autoinflammatory condition)	6	Positive - immunocompromised patient treated with foscarnet
11	HHV6	7 years	Repeat following ME panel positive for VZV	Yes (autoinflammatory condition)	2	None
12	HHV6	1 year	Fever and seizure vs rigors	No	1	None
13	HHV6	9 months	Fever and irritability	No	2	None
14	HHV6	2 years	Fever and irritability	No	1	None
15	HHV6	3 years	Status epilepticus in setting of subtherapeutic antiepileptic	No	<1	None
16	HHV6	1 year	Fever and seizure	No	2	None
17	HHV6	15 years	Altered mental status and leg numbness/tingling	No	65	None
18	HHV6	1 year	Shock with multiorgan disfunction and necrotizing encephalopathy in previously healthy child	No	1	Unclear - significance of HHV6 not clear but treated with ganciclovir. No other infectious cause identified on other testing.
19	HHV6	1 year	Fever and seizure	No	4	None
20	HHV6	8 months	Seizure	No	2	None
21	HHV6	<1 week	Poor feeding and lethargy	No	Not performed	None
22	HHV6	2 weeks	Fever	No	8	None
23	HHV6	9 months	Seizure	No	2	None
24	HHV6, *Streptococcus pneumoniae*	7 years	Fever, headache, and emesis	No	235	Negative - Received several days ganciclovir
25	HHV6, *Streptococcus pneumoniae*	2 years	Fever and emesis	No	323	None
26	HSV1	15 years	Seizure, history of neonatal HSV infection	No	113	None - dedicated HSV1 PCR also positive, treated for CNS HSV
27	HSV1	3 years	Cranial nerve palsy	No	226	Positive - treated for CNS HSV (dedicated HSV PCR not sent)
28	HSV1	7 months	Seizure, history of neonatal HSV infection	No	10	None - dedicated HSV1 PCR also positive, treated for CNS HSV
29	HSV2	8 years	fever, headache	Yes (s/p SOT)	456	Positive - treated for CNS HSV (dedicated HSV PCR not sent)
30	HSV2	11 months	Fever and seizure	No	205	Negative - dedicated HSV2 PCR negative, received brief course acyclovir until deemed false positive, ultimately received non-infectious diagnosis
31	HSV2	3 weeks	Fever, seizure, shock	No	155	None - dedicated HSV2 PCR also positive, treated for CNS HSV

A positive herpes virus result was determined to have had a positive impact if there was consensus among the providers involved in the patient’s care that it represented a true infection and allowed for addition of targeted therapy, determination of the need to continue therapy that had been started empirically, or discontinuation of a therapy that had been started empirically. It was determined to have a negative impact if there was ultimately consensus from providers involved in the patient’s care that it did not explain the symptoms for which a lumbar puncture was performed but additional medications were given or tests performed because of the positive test. It was characterized as unclear impact if there was not ultimately consensus about the significance of the result among the providers who saw the patient. There was considered to be no impact if the test result did not change clinical management or the diagnosis had been made by another test sent at the same time.

N.B. Samples 4/10/11 and 8/9 were from the same patients at different time points

CMV,cytomegalovirus; *E. coli,Escherichia coli;* HHV6,human herpes virus 6; CNS,central nervous system; HSV,herpes simplex virus; PCR,polymerase chain reaction; SOT,solid organ transplant; VZV,varicella zoster virus.

## Discussion

We found that at our institution, management of immunocompetent children rarely changed when herpesviruses were detected on the ME panel. The widespread commercial availability of molecular tests has led to significant variation in utilization when optimal use case scenarios are not well defined.^9–11^ Our ME panel findings highlight the potential consequences of test overuse outlined by the Society for Healthcare Epidemiology of America: overdiagnosis of organisms not causing true disease, possible misdiagnosis if providers anchor on a positive result from this test, and excess cost burdens on the healthcare system.^12^

This work does have multiple limitations. Significantly, we did not evaluate the impact of negative tests or the psychological impact of positive tests that did not change clinical management. Given the retrospective nature of this study, we were unable to reach definitive conclusions about the impact of positive herpesvirus results on antimicrobial management. This study was not designed to evaluate the test performance characteristics of the ME panel, and our use of impact on clinical management as our main outcome does introduce the possibility of bias into the interpretation of the test impact. However, this measure still has value as it reflects the real-world impact of the test. Our findings are also likely less generalizable to other sites with strong stewardship strategies in place for this test, however the impact of a false positive result remains.

At our site, de-implementation of ME panels represent a significant opportunity for diagnostic stewardship,^13^ and we are actively pursuing quality improvement work to do so. We urge other sites with unrestricted use of multiplex panels to similarly consider potential downsides of this broad testing approach and seek out opportunities to optimize use of multiplex panel testing.

## Data Availability

To protect patient privacy, data beyond those provided in this manuscript are not available for dissemination.
